# T2* quantification using multi-echo gradient echo sequences: a comparative study of different readout gradients

**DOI:** 10.1038/s41598-023-28265-0

**Published:** 2023-01-20

**Authors:** Seonyeong Shin, Seong Dae Yun, N. Jon Shah

**Affiliations:** 1 Institute of Neuroscience and Medicine 4, Forschungszentrum Jülich, INM-4, 52428 Jülich, Germany; 2 RWTH Aachen University, Aachen, Germany; 3 Institute of Neuroscience and Medicine 11, JARA, Forschungszentrum Jülich, INM-11, Jülich, Germany; 4 JARA-BRAIN-Translational Medicine, Aachen, Germany; 5 Department of Neurology, RWTH Aachen University, Aachen, Germany

**Keywords:** Magnetic resonance imaging, Magnetic resonance imaging

## Abstract

To quantify T2*, multiple echoes are typically acquired with a multi-echo gradient echo sequence using either monopolar or bipolar readout gradients. The use of bipolar readout gradients achieves a shorter echo spacing time, enabling the acquisition of a larger number of echoes in the same scan time. However, despite their relative time efficiency and the potential for more accurate quantification, a comparative investigation of these readout gradients has not yet been addressed. This work aims to compare the performance of monopolar and bipolar readout gradients for T2* quantification. The differences in readout gradients were theoretically investigated with a Cramér-Rao lower bound and validated with computer simulations with respect to the various imaging parameters (e.g., flip angle, TR, TE, TE range, and BW). The readout gradients were then compared at 3 T using phantom and in vivo experiments. The bipolar readout gradients provided higher precision than monopolar readout gradients in both computer simulations and experimental results. The difference between the two readout gradients increased for a lower SNR and smaller TE range, consistent with the prediction made using Cramér-Rao lower bound. The use of bipolar readout gradients is advantageous for regions or situations where a lower SNR is expected or a shorter acquisition time is required.

## Introduction

The distinct and detailed tissue contrast associated with MR images is dependent on both intrinsic and extrinsic parameters. Unlike extrinsic parameters, which can be manipulated depending on the sequence/scanner used, intrinsic parameters (in the absence of contrast agent injection) cannot be easily changed and are inherent to different tissue types. Consequently, by quantifying these intrinsic tissue parameters, such as relaxation times, MR images can be more effectively interpreted. This allows the direct comparison of subject data and also provides indices of disease severity^1–5^.

The measurement of T2* relaxation times using MRI has now become common practice due to the improved SAR efficiency and faster acquisition afforded by the development of multi-echo gradient echo (GRE) sequences^6–8^. Multi-echo GRE sequences can be divided into two types according to the readout gradients: monopolar and bipolar. In monopolar readouts, fly-back gradients are placed between adjacent echoes to maintain readout gradient polarity and to ensure phase consistency^9^. All echoes are acquired with the same readout gradient polarity. However, this increases the echo spacing time, ΔTE, resulting in a reduction of the number of echoes, *N*_*e*_, for a given readout time. Additionally, if fly-back gradients of short duration and large magnitude are employed to minimise the influence on ΔTE, the potential for eddy current generation is increased. In bipolar readouts, multiple echoes are obtained with alternating readout gradient polarities, as is the case in an echo-planar imaging (EPI) readout^10–12^. The use of bipolar readout gradients shortens the ∆TE, leading to a reduction of repetition time (TR) or an increase in the *N*_*e*_ within a fixed scan time. In statistical estimation, a larger number of observations leads to increased precision^13^. Thus, theoretically, bipolar readouts can provide a more reliable T2* than monopolar readouts. A sequence using the bipolar readout, termed QUTE, was demonstrated before the monopolar version^11,12^. Subsequently, the simpler and more eddy-current robust monopolar sequence was developed.

Despite these significant advantages, most studies have opted to use monopolar readout due to the challenges associated with implementing artefact-free and robust bipolar readout^14,15^. For example, magnetic field inhomogeneity could induce geometric distortions along the readout direction^16,17^, phase errors can arise from the eddy currents or hardware imperfection^18,19^, and the frequency response of the MR receiver system can also produce asymmetric amplitude modulation^20^. As the geometric distortion, phase, and amplitude errors appear in opposite directions in every other echo, the use of bipolar readout is much more demanding, and achieving accurate quantification results can be difficult. However, geometric distortions induced by global field inhomogeneities can be suppressed under well-shimmed conditions^17^, and amplitude and phase errors can be compensated for with various correction methods^18,21–23^.

Given this possible variation in results and the potential impact on the accuracy and precision of quantitative T2* measurements, this work describes a practical implementation of bipolar readout and characterises the quantitative results obtained in terms of precision. The precision of monopolar and bipolar readouts was investigated using computer simulation and was verified with a Cramér-Rao lower bound (CRLB) under various signal-to-noise ratio (SNR) conditions. Phantom and in vivo experiments were then conducted at 3 T with respect to the flip angle, α, and parallel imaging acceleration factor, R. The performance of the readout gradients was also compared as a function of various echo time (TE) ranges and ∆TE by retrospectively adjusting the *N*_*e*_. To our knowledge, this is the first time that T2* measurements using monopolar and bipolar readout gradients have been characterised in such detail, paving the way for the uptake of bipolar readouts.

## Methods

### Computer simulation datasets

Computer simulations were conducted to investigate the performance of the readout gradients. Schematic diagrams of multi-echo GRE sequences are shown in Fig. S1 in the Supplementary Information. The monopolar readout acquires multi-echo data with only positive (or only negative) readout gradients. In contrast, both polarities of the readout gradients are utilised to acquire data when using bipolar readout. The TEs are determined by the time required before signals are acquired (*T*_*prep*_), which includes RF-pulse duration, slice-selection, phase-encoding, and readout dephasing gradients, the time required to obtain each echo (*T*_*acq*_) and the time needed for gradient ramp up and down (*T*_*ramp*_). In monopolar readout, the time required for fly-back gradients (*T*_*fb*_) also affects the TE. The TEs for each readout gradient can be written as:

1 $$\mathrm{monopolar}: T{E}_{n}={T}_{prep}+2n-1\left(\frac{{T}_{acq}}{2}+{T}_{ramp}\right)+\left(n-1\right){T}_{fb},$$

and

2 $$\mathrm{bipolar}: T{E}_{n}={T}_{prep}+2n-1\left(\frac{{T}_{acq}}{2}+{T}_{ramp}\right),$$

where *n* = [1, 2, …, *N*_*e*_] is the echo index. *N*_*e*_ is the total number of echoes.

The signal from a spoiled multi-echo GRE sequence can be expressed as:

3 $$s\left(T{E}_{n}\right)={M}_{0}\left(\frac{\mathit{sin}\left(\alpha \right)\left(1-{e}^{-TR/T1}\right)}{1-\mathit{cos}(\alpha ){e}^{-TR/T1}}\right){e}^{-T{E}_{n}/T{2}^{*}}{e}^{1i\left({\varphi }_{0}+2\pi \Delta fT{E}_{n}\right)}+\varepsilon \left(0,{\sigma }^{2}\right),$$

where M_0_ is the spin density, α is a flip angle, TR is the repetition time, T1 is the longitudinal relaxation time and T2* is the transverse relaxation time. The parameters *φ*_*0*_ and ∆*f* represent the initial phase and off-resonance frequency, respectively. The parameter ε is the additive white Gaussian noise with a zero-mean and variance of σ^2^. Neither phase nor amplitude modulation were included in Eq. (3) as an ideal situation was assumed.

Simulation datasets for monopolar and bipolar readouts were created using Eqs. (1–3). For a given TR, the spacing between the echoes, ∆TE, can be reduced by increasing the receiver bandwidth (BW), thereby increasing the *N*_*e*_ for T2* quantification. However, increasing the BW results in a decrease in SNR. In order to explore the relationships between BW, ∆TE, *N*_*e*_ and SNR, the *T*_*acq*_ and ε(0,σ^2^) were modelled as follows. The *T*_*acq*_ depends on the number of samples collected for each echo (*N*_*x*_) and the BW. Thus, *T*_*acq*_ was modelled as a rounding up of *N*_*x*_/BW. The σ of Gaussian noise was simulated by^24^:

4 $$\sigma ={\sigma }_{0}\sqrt{BW/B{W}_{0}},$$

where σ_0_ is the standard deviation of the image noise with a receiver bandwidth of BW_0_.

**Figure 1:**
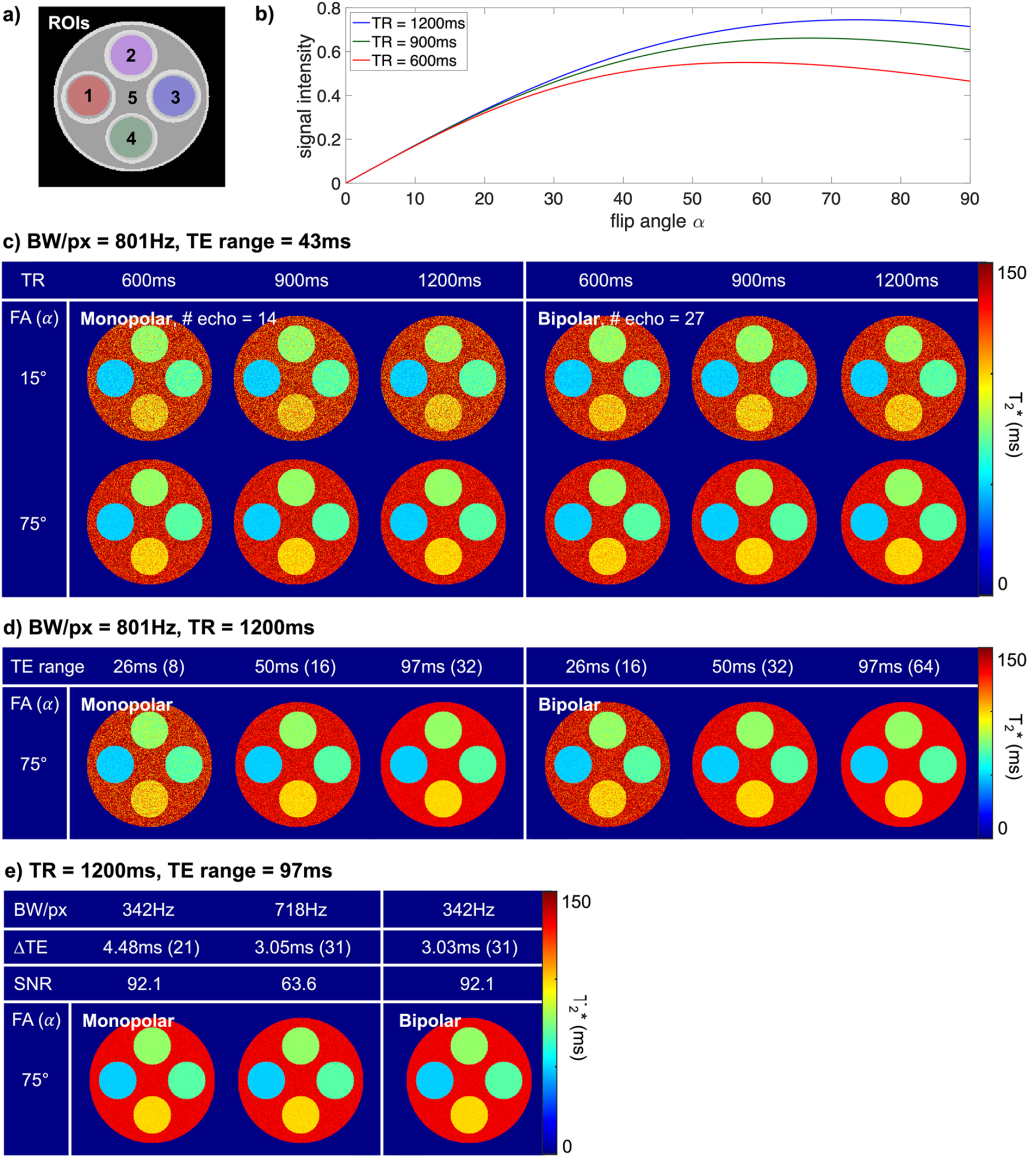
Computer simulation results. (**a**) Location of ROIs. (**b**) Plot of the signal intensities with respect to α in ROI 3. The TRs of 600 ms, 900 ms and 1200 ms are denoted by red, green, and blue lines, respectively. (**c**) T2* maps with respect to the TR. The ∆TE was 2.92 ms for the monopolar readouts and 1.47 ms for the bipolar readouts with a BW/px of 801. To solely observe the effect of SNR, the TE range was limited to 43 ms for all TRs. (**d**) T2* maps with respect to the TE range. As the TE range increases, the variation in the T2* map decreases due to the increased number of echoes. (**e**) T2* maps with respect to BW. The ∆TE decreases with an increase in the BW, allowing more echoes to be collected in a given TE range, but reducing SNR.

Three simulations were performed to investigate the effects of (1) α and TR (i.e., SNR under the fixed BW), (2) TE range, and (3) BW on the quantification of T2*. The simulation phantom was designed to resemble the phantom used in our experiments. It consisted of five regions, each of which was designed to have a T2* of 49 ms, 78 ms, 69 ms, 99 ms, and 133 ms, and an accompanying T1 of 671 ms, 959 ms, 878 ms, 1134 ms and 1372 ms, respectively (Fig. 1a). The imaging parameters used in each simulation are summarised in Table S1. For the first simulation, the BW and TE range were fixed to 192.24 kHz and 43 ms. Datasets were generated with three different values of α and TR. For the second simulation, the BW, TE range and TR were fixed to 192.24 kHz, 97 ms and 1200 ms. Datasets were created with α values of 15°, 35° and 75°. After creating a total of three datasets for each readout, subsampling was performed along the TE dimension. The first *n* echoes out of the echoes created were selected and used for T2* quantification. The parameter *n* was increased from 3 to 32 for monopolar readouts and from 5 to 64 for bipolar readouts (i.e., we can observe the situation where the TE range was increased from around 10–97 ms). As an additional experiment, every *n-th* echo out of the echoes created was selected. The datasets with the same TE range were then compared to assess the effect of ∆TE on T2* quantification. The parameter *n* was increased from 2 to 31 for monopolar readouts and from 2 to 63 for bipolar readouts, respectively. For the third simulation, the parameters α, TR and TE range were fixed to 75°, 1200 ms and 97 ms. Datasets were simulated with three different BWs.

### Phantom production

A spherical phantom was constructed to compare the readout gradients for various T2* relaxation times. The phantom comprised four small flasks, each of which was filled with a different concentration of manganese (II) chloride (MnCl_2_). The concentrations were 0.088, 0.128, 0.113, 0.183, and 0.064 mM, and were created to mimic the T2 of brain tissues (i.e., grey matter, white matter, spinal cord, bone marrow, and marrow fat) at 3 T, respectively^25,26^.

### Phantom experiment datasets

Phantom experiments were performed at a 3 T PRISMA MR scanner (Siemens Healthineers, Erlangen, Germany) with a 20-channel head/neck coil. As in the computer simulation, the differences in readout gradients were investigated for (1) α and TR, (2) TE range, (3) R, and (4) BW. The imaging parameters were set to have a scan time of 5 min, without any acceleration, using in-house developed GRE sequences: FOV = 240 × 240 mm^2^, matrix = 240 × 240, slice thickness = 1 mm, *N*_*s*_ = 11, BW/px = 801 Hz, R = 1, TE_1_ = 3.84 ms, ∆TE = 2.92 ms for monopolar readout and 1.47 ms for bipolar readout, and 10 preparation scans. The readout gradients were set to have a maximum number of echoes in a given condition, and the amplitude of the fly-back gradients was also set to have a maximum value when using monopolar readouts. As a first experiment, datasets with an α of 75°, 35°, and 15° were acquired for the TRs of 600 ms, 900 ms and 1200 ms (i.e., a total of nine datasets for each readout). This was done to observe the results of T2* quantification with respect to the SNR. The achievable *N*_*e*_ increases as the TR increases. However, to remove the effect of an increase in the *N*_*e*_, datasets with the TR of 900 ms and 1200 ms were retrospectively subsampled along the TE dimension to have the same TE range and *N*_*e*_ in all datasets. The TE range was 43 ms, and the *N*_*e*_ was 14 for monopolar and 27 for bipolar readouts. In a subsequent experiment, datasets with an α of 35° and 15° were obtained for the TR of 1200 ms (i.e., a total of four datasets for each readout) and compared to the dataset with an α of 75°. When the TR was 1200 ms, the maximum TE range was 97 ms. The *N*_*e*_ was 32 for monopolar and 64 for bipolar readouts. After acquiring the datasets, the first *n* echoes out of the 32 (monopolar) and 64 (bipolar) echoes acquired were retrospectively chosen as in the computer simulation. The T2* values were then calculated with the selected echoes and compared to observe the performance of the readout gradients under the various TE ranges. In addition, every *n-th* echo out of the echoes acquired was retrospectively selected. From the generated datasets, those with the same TE range were compared to investigate the effect of the ∆TE. In a third experiment, the R was prospectively varied from 2 to 3 for the datasets with an α of 75° and TR of 1200 ms (i.e., acquire two datasets for each readout). The 24 reference lines were obtained separately for the GRAPPA reconstruction. As a fourth experiment, datasets with the BW/px of 718 Hz, 613 Hz and 342 Hz were additionally produced (i.e., a total of six datasets for each readout) to explore the effects of BW. The imaging parameters for the fourth experiment were as follows: FOV = 240 × 240 mm^2^, matrix = 240 × 240, slice thickness = 1 mm, *N*_*s*_ = 11, α = 75°, TR = 1200 ms, R = 1, TE range = 97 ms, and 10 preparation scans. The TE_1_s were 3.9 ms, 4 ms and 4.62 ms for BW/px of 718 Hz, 613 Hz and 342 Hz, respectively. The ∆TEs were 3.03 ms, 3.2 ms and 4.39 ms for monopolar readouts, and 1.6 ms, 1.8 ms and 3.03 ms for bipolar readouts, respectively.

### In vivo experiment datasets

Datasets from healthy volunteers were acquired with the following parameters: FOV = 240 × 240 mm^2^, matrix = 240 × 240, slice thickness = 1 mm, TR = 1200 ms, *N*_*s*_ = 11, BW/px = 801 Hz, TE_1_ = 3.84 ms, ∆TE = 2.92 ms for monopolar (*N*_*e*_ = 32) and 1.47 ms for bipolar readouts (*N*_*e*_ = 64) and 10 preparation scans. To keep the total acquisition time as short as possible, only datasets with an α of 76° and 24° were acquired. The R was prospectively changed from 1 to 3 for 76° to compare the results with respect to the SNR (i.e., a total of four datasets for each readout). The 24 reference lines were collected separately for the GRAPPA reconstruction. The *N*_*e*_ was retrospectively adjusted, as was the case in the phantom experiments. The first *n* echoes out of the 32 (monopolar) and 64 (bipolar) echoes acquired were selected and compared to investigate the performance of the readout gradients under the various TE ranges. In addition, every *n-th* echo was collected, and the datasets with the same TE range were compared to observe the effects of ∆TE.

This study was approved by the local institutional review board (RWTH Aachen University, approval number EK 266/09) and performed in accordance with relevant guidelines and regulations. Informed consent was obtained from all subjects prior to the experiments.

### Data analyses

For experimental data, the individual coil images were reconstructed by applying 2D Fourier transform without any additional k-space filtering, and the resultant multi-channel images were combined with an adaptive combine method^27^. When parallel imaging was applied, the individual coil image was reconstructed using a 5 × 4 kernel. To quantify T2*, magnitude fitting was performed in each voxel. The influences of α, TR, and T1 (i.e., the second term in Eq. 3) can be seen as a multiplication of a constant term, and the phase information (i.e., the fourth term in Eq. 3) vanishes when the magnitude image is treated. Thus, mono-exponential decay was employed for the calculation of T2*. The Rician noise distribution was also considered^28,29^. The noise variance was estimated from four ROIs, consisting of 300 voxels, at the corners of the noise-only background. When the bipolar readouts were used, amplitude modulation was also modelled as an exponential function and corrected for experimental datasets^22^. The spin density and T2* values were first calculated from all echoes, and then the exponential terms multiplied by the odd and even echoes were computed using the difference between the measured values and the synthesised values. This procedure was repeated iteratively.

The mean and standard deviation of the calculated T2*s were evaluated for each ROI. The ROIs were manually determined for computer simulations and phantom experiments. The segmentation results obtained using FSL’s FAST and FIRST algorithms^30,31^ were used as masks for the in vivo experiments. For the phantom experiments where true T2* was unknown, the mean values were compared based on the values obtained with a relatively higher SNR (i.e., α of 75° without any acceleration). The mean values of the in vivo data were compared to those reported in other literature^3,32,33^. The measured standard deviations were also compared with the theoretically predicted values using a CRLB. A Rician noise distribution was considered for the calculation of the Fisher information matrix^34^.

## Results

Figure 1 shows the representative results of the computer simulation. Figure 1a shows the location of the regions of interest (ROIs). Figure 1b shows the relative transverse magnetisation level of ROI 3 (T2* = 69 ms, T1 = 878 ms) with respect to the α and TR. In Fig. 1c–e, the left and right figures show T2* maps derived from the use of monopolar and bipolar readouts, respectively. Figure 1c shows that the variation in the T2* map increases as the SNR decreases (i.e., as the α and TR decrease). The T2* map was hardly affected by the changes in the TR at a lower α (i.e., 15°), since the SNR remained almost the same (Fig. 1b). Figure 1d depicts the changes in the T2* map with respect to the TE range when the TR was kept constant. An increase in the TE range enables more echoes to be acquired; the maximum number of echoes achieved with the given TE range is indicated in parentheses. Under the given SNR condition (i.e., fixed α and TR), variation in the T2* map decreases as the TE range increases. The T2* maps from the bipolar readouts depicted in Fig. 1c,d show less variation than those from monopolar readouts. This is particularly significant with a lower SNR and shorter TE range. Figure 1e shows the effects of the BW. The ∆TE for each BW/px and the corresponding number of echoes are indicated at the top of the figures. The SNR is the value measured in the first echo image. The bipolar readout can acquire the same number of echoes as the monopolar readout while decreasing BW and improving SNR (e.g., 718BW/px for monopolar and 342BW/px for bipolar). In this case, a T2* map from a bipolar readout can achieve less variation. The mean and standard deviation of T2* values for each simulation experiment are presented in Table 1. The number in parentheses is the standard deviation predicted by the CRLB.

**Table 1:** Mean and standard deviation of the calculated T2*s in the computer simulation with respect to the (a) α and TR (b) α and TE range (c) ∆TE and (d) BW/px.

TR		600 ms	900 ms	1200 ms
(a) BW/px = 801 Hz				
15°	Mono	69.95 ± 8.45 (7.76)	69.83 ± 8.17 (7.64)	69.65 ± 7.77 (7.58)
	Bipolar	69.66 ± 6.11 (5.75)	69.45 ± 5.88 (5.66)	69.38 ± 5.68 (5.62)
35°	Mono	69.07 ± 4.05 (4)	69.11 ± 3.77 (3.72)	69.36 ± 3.63 (3.59)
	Bipolar	69.15 ± 2.96 (2.97)	69.06 ± 2.74 (2.76)	69.1 ± 2.65 (2.66)
75°	Mono	69.01 ± 3.67 (3.52)	69.13 ± 2.88 (2.84)	69.01 ± 2.43 (2.51)
	Bipolar	69.08 ± 2.58 (2.61)	69.08 ± 2.13 (2.1)	69.02 ± 1.84 (1.86)

TE range		26 ms	52 ms	97 ms
(b) BW/px = 801 Hz, TR = 1200 ms				
15°	Mono	73.05 ± 20.97 (15.48)	69.47 ± 6.65 (6.48)	69.01 ± 3.31 (3.26)
	Bipolar	70.68 ± 11.68 (10.93)	69.28 ± 4.64 (4.6)	69.05 ± 2.29 (2.33)
35°	Mono	69.94 ± 7.98 (7.33)	69.28 ± 3.1 (3.07)	69.09 ± 1.56 (1.54)
	Bipolar	69.25 ± 5.35 (5.17)	69.07 ± 2.16 (2.18)	69 ± 1.09 (1.1)
75°	Mono	69.42 ± 5.22 (5.14)	69.06 ± 2.12 (2.15)	69.01 ± 1.05 (1.08)
	Bipolar	69.03 ± 3.67 (3.63)	69.03 ± 1.53 (1.53)	69.02 ± 0.77 (0.77)

∆TE (mono/bipolar)		43.8/44.1 ms	17.52/17.64 ms	5.84/5.88 ms
(c) BW/px = 801 Hz, TR = 1200 ms, TE range = 92 ms				
75°	Mono	69.07 ± 2.91 (2.83)	68.94 ± 2.23 (2.23)	69.01 ± 1.49 (1.49)
	Bipolar	69.08 ± 2.84 (2.83)	69.03 ± 2.22 (2.23)	68.99 ± 1.46 (1.5)

BW/px		342 Hz	613 Hz	718 Hz
(d) TR = 1200 ms, TE range = 97 ms				
∆TE (mono/bipolar)		4.48/3.03 ms	3.27/1.82 ms	3.05/1.6 ms
# of echoes (mono/bipolar)		21/31	29/52	31/59
75°	Mono	69.02 ± 0.86 (0.87)	69.04 ± 0.98 (0.99)	69 ± 1.04 (1.04)
	Bipolar	68.99 ± 0.72 (0.72)	69.03 ± 0.76 (0.75)	69.01 ± 0.76 (0.76)

The values were measured in ROI 3 (Fig. 1a). The number in the parentheses shows the predicted standard deviation using a CRLB. As with the visual observation, standard deviation increases as the α, TR, and the TE range decrease. (a and b) The difference between the two readouts becomes larger for a lower SNR and shorter TE range. (c) The ‘std diff’ is substantially smaller when the ∆TEs from each readout are similar. (d) By decreasing the BW, the bipolar readout can achieve the same number of echoes as the monopolar readout but with higher precision. When the BW/px of monopolar and bipolar readouts are 718 Hz and 342 Hz, respectively, both readouts acquire 32 echoes. However, bipolar readouts provide a smaller standard deviation (i.e., 0.72 ms) compared to monopolar readouts (i.e., 1.04 ms).

**Figure 2:**
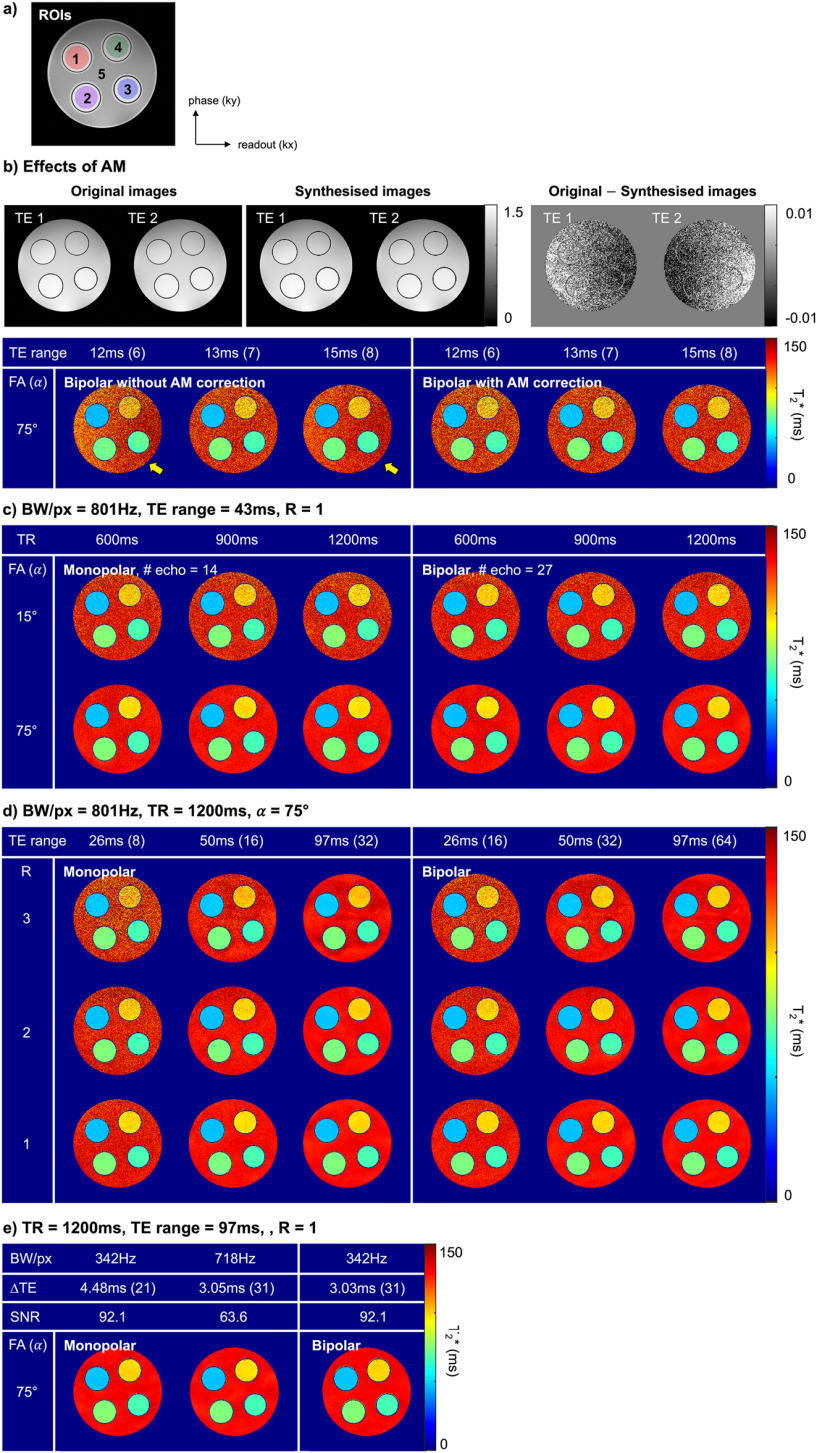
Phantom experiment results. (**a**) Location of ROIs. (**b**) Effects of the AM modulation. Imaging parameters: BW/px = 801, TR = 1200 ms, α = 75°, ∆TE = 1.47 ms, and R = 1. The synthesised images in the first row are created from a dataset with a TE range of 97 ms. Before amplitude modulation correction, the left and right parts of the object show slightly different T2* values. The T2* was stably computed over the entire region after amplitude modulation correction. (**c**–**e**) T2* maps with respect to the (**c**) α and TR, (**d**) R and TE range, and (**e**) BW. The ∆TE was 2.92 ms for the monopolar readouts and 1.47 ms for the bipolar readouts under the BW/px of 801. The variation in the T2* map increases as the α and TE range decrease and the R increases. An increase in the BW reduces the ∆TE, allowing more echoes to be collected within the same scan time, but with a reduced SNR.

Figure 2 shows the results of the phantom experiments. Figure 2a shows the location of ROIs. Figure 2b displays the effects of the amplitude modulation. The first row displays the first and second echo images taken with bipolar readout, the synthesised images created using the fitted parameters without amplitude modulation, and the difference images between the acquired and synthesised images. The images were produced from a dataset with a TE range of 97 ms. It is challenging to visually discern the amplitude modulation in the images obtained. However, the difference images clearly show that amplitude modulation produced different signal intensity modulation along the readout direction. In the second row, the left and right figures are the T2* maps from bipolar readout when the TE range is relatively short. The figures on the right are the T2* maps following amplitude modulation correction. The *N*_*e*_ achieved with the given TE range is indicated in parentheses at the top of the figure. The amplitude modulation makes the difference in T2* along the readout direction, as indicated by yellow arrows. However, it can be compensated by amplitude modulation correction. In Fig. 2c–e, the T2* maps from bipolar readout followed amplitude modulation correction. Figure 2c shows that the variation in the T2* map increases as the α and TR decrease. The T2* map from the bipolar readout gives a smaller variation compared to that from the monopolar readout under the same conditions, especially with a lower SNR (i.e., lower α and shorter TR). Figure 2d shows that the variation in the T2* map increases with an increase in the R and a decrease in the TE range. The difference between the T2* maps from the two readouts becomes larger for a higher R and shorter TE range. Figure 2e shows the changes in the T2* map with respect to the BW. The bipolar readouts can acquire the same number of echoes as the monopolar readouts while increasing the SNR with a lower BW. Table 2 shows the mean, standard deviation, and CRLB values for each phantom experiment.

**Table 2:** Mean, standard deviation and relative difference of the standard deviation with respect to the (a) α and TR (b) α and TE range (c) R and TE range (d) ∆TE and (e) BW/px in phantom experiments.

TR		600 ms	900 ms	1200 ms
(a) BW/px = 801 Hz, TE range = 43 ms, R = 1				
15°	Mono	66.91 ± 4.19 (3.93)	66.59 ± 4.04 (3.88)	66.63 ± 4.09 (3.85)
	Bipolar	66.72 ± 3.27 (2.9)	66.51 ± 3.22 (2.88)	66.27 ± 3.19 (2.85)
35°	Mono	66.39 ± 2.23 (1.87)	66.34 ± 2.14 (1.77)	66.49 ± 2.18 (1.74)
	Bipolar	66.66 ± 1.91 (1.37)	66.4 ± 1.88 (1.31)	66.5 ± 1.87 (1.28)
75°	Mono	66.58 ± 1.75 (1.36)	66.06 ± 1.62 (1.17)	66.46 ± 1.58 (1.11)
	Bipolar	66.74 ± 1.61 (1.01)	66.57 ± 1.54 (0.88)	66.08 ± 1.5 (0.81)

TE range		26 ms	52 ms	97 ms
(b) BW/px = 801 Hz, TR = 1200 ms, R = 1				
15°	Mono	67.19 ± 8.2 (7.94)	66.59 ± 3.61 (3.29)	66.12 ± 2.34 (1.66)
	Bipolar	66.46 ± 5.66 (5.55)	66.3 ± 2.82 (2.34)	66.15 ± 1.97 (1.18)
35°	Mono	66.44 ± 3.69 (3.52)	66.54 ± 2.07 (1.49)	66.6 ± 1.76 (0.76)
	Bipolar	66.5 ± 2.72 (2.48)	66.52 ± 1.82 (1.05)	66.56 ± 1.62 (0.54)
75°	Mono	66.59 ± 2.38 (2.26)	66.48 ± 1.54 (0.95)	66.69 ± 1.48 (0.49)
	Bipolar	66.08 ± 1.85 (1.6)	66.11 ± 1.52 (0.67)	66.29 ± 1.42 (0.34)
(c) BW/px = 801 Hz, TR = 1200 ms, α = 75°				
R = 3	Mono	67.46 ± 6.38 (6.12)	66.73 ± 2.86 (2.52)	66.99 ± 1.95 (1.29)
	Bipolar	67.44 ± 4.44 (4.33)	67.13 ± 2.35 (1.82)	67.4 ± 1.74 (0.93)
R = 2	Mono	66.99 ± 3.53 (3.43)	66.85 ± 1.89 (1.44)	67.22 ± 1.6 (0.74)
	Bipolar	67 ± 2.59 (2.41)	66.94 ± 1.73 (1.02)	67.24 ± 1.5 (0.53)

∆TE (mono/bipolar)		43.8/44.1 ms	17.52/17.64 ms	5.84/5.88 ms
(d) BW/px = 801 Hz, TR = 1200 ms, TE range = 92 ms, R = 1				
75°	Mono	66.68 ± 1.73 (1.28)	66.72 ± 1.62 (1.00)	66.7 ± 1.53 (0.67)
	Bipolar	66.31 ± 1.72 (1.26)	66.31 ± 1.61 (0.99)	66.29 ± 1.52 (0.66)

BW/px		342 Hz	613 Hz	718 Hz
(e) TR = 1200 ms, TE range = 97 ms, R = 1				
75°	Mono	66.83 ± 1.44 (0.38)	66.68 ± 1.45 (0.44)	66.68 ± 1.46 (0.47)
	Bipolar	66.49 ± 1.39 (0.33)	66.66 ± 1.43 (0.34)	66.89 ± 1.39 (0.34)

The values were calculated in ROI 3 (Fig. 2a). The numbers in parentheses are the standard deviations predicted from CRLB. As in the computer simulation, the standard deviation increases as the α, TR and TE range decrease. The standard deviation also increases with an increase in the R. (a-c) The difference between the two readouts tends to increase at a lower α, shorter TR, shorter TE range, and lower R. (d) When the TEs from each readout are similar and have the same BW/px, the monopolar and bipolar readouts provide similar precision. (e) When the monopolar and bipolar readouts have the BW/px of 718 and 342 Hz, respectively, both readouts achieve 31 echoes in the same scan time. However, the bipolar readout can achieve a smaller standard deviation (i.e., 1.39 ms) than the monopolar readout (i.e., 1.46 ms) with an increased SNR.

**Figure 3:**
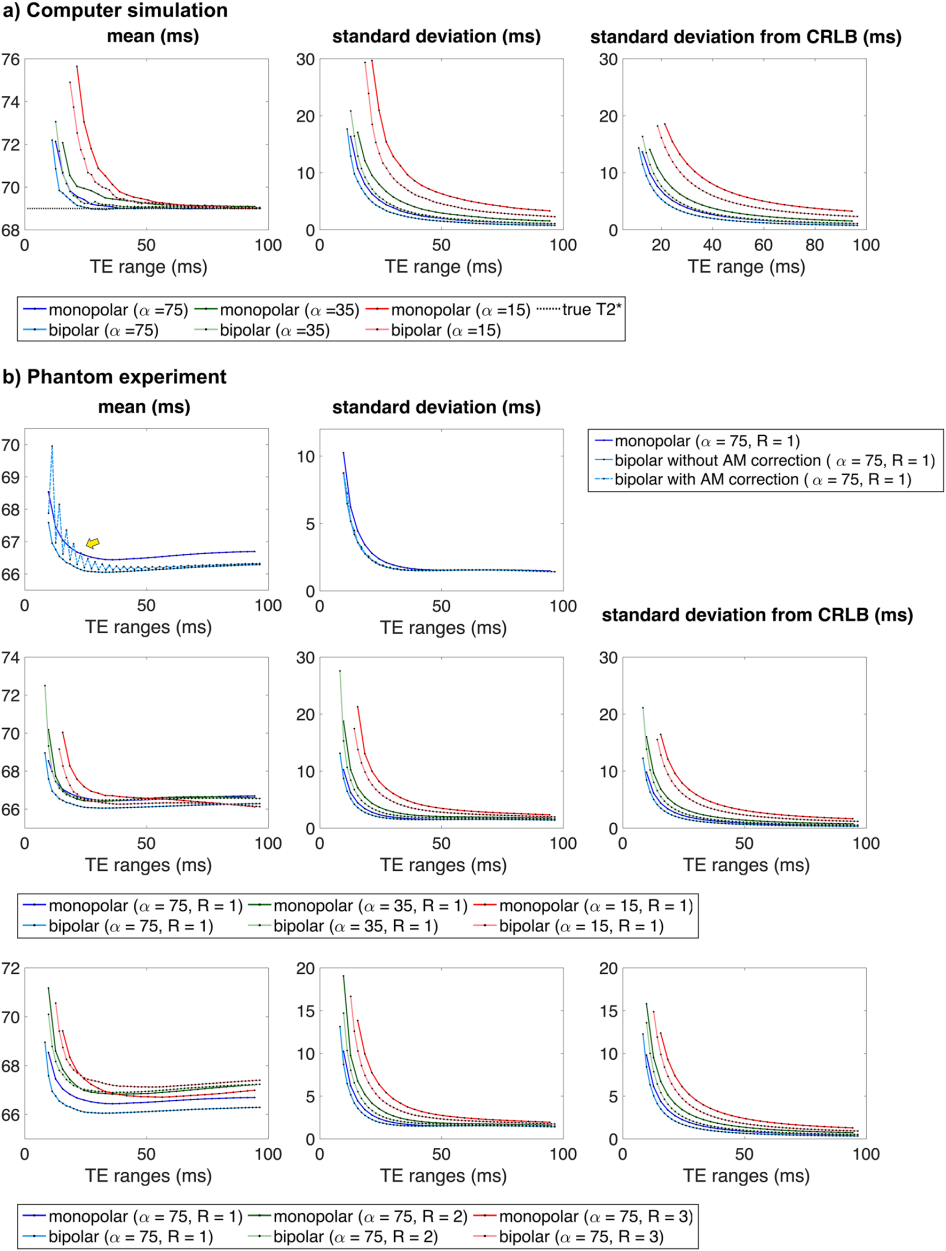
Plots of the mean and standard deviations as a function of the TE range. The mean and standard deviations were measured in ROI 3. Imaging parameters: BW/px = 801, ∆TE = 2.92 ms for monopolar and 1.47 ms for bipolar, TR = 1200 ms. (**a**) Computer simulation results. (**b**) Phantom experiments results. The first row shows the effects of amplitude modulation. In the case of bipolar readout, the fluctuations in the mean value can be removed by an amplitude modulation correction. In the second and third rows, the changes in mean and standard deviations are displayed with respect to the α and R. As the SNR decreases (i.e., as the α decreases and R increases), the mean and standard deviation tend to increase.

The mean, standard deviation, and CRLB values are also plotted as a function of the TE range in Fig. 3. Figure 3a displays the results of computer simulations. The results where α is 75°, 35°, and 15° are denoted by the blue, green, and red lines, respectively. The results from the monopolar and bipolar readouts are depicted with dark and light colours, respectively. In the mean plot, the true T2* is indicated by a dotted black line. The mean T2* decreases as the TE range increases and eventually reaches a convergence value (i.e., true T2*). The TE range required to reach the convergence value increases as the α decreases. For instance, the mean T2* of the monopolar readout converges to a true value near the TE range of 30 ms for 75° and 70 ms for 15°. When the TE range is sufficiently long, the mean values from the bipolar and monopolar readouts are almost the same. However, with a shorter TE range, the mean from the bipolar readout tends to be closer to the true value than that from the monopolar readout. The standard deviation decreases with the increase in the TE range and α. The measured standard deviations (2nd column) showed the same tendency as predicted by CRLB (3rd column). The results from the bipolar readout had lower standard deviations compared to those from the monopolar readout, regardless of the TE ranges. Specifically, the difference between the two readouts became larger for a lower α and shorter TE range. Figure 3b shows the results of phantom experiments. The first row shows the effects of amplitude modulation. The results from the bipolar readout before and after amplitude modulation correction are depicted by different types of lines. In the case where bipolar readouts are utilised without amplitude correction, the mean T2* fluctuates, as indicated by the yellow arrow. These fluctuations are eliminated by an amplitude modulation correction. For standard deviation, the correction of amplitude modulation did not have a significant impact on its value. The second and third rows display the changes in the value with respect to the α and R, respectively. In each plot, the different α or Rs are indicated by blue, green, and red lines. The results obtained using monopolar and bipolar readouts are denoted by dark and light colours, respectively. The mean and standard deviation tend to decrease as the TE range increases. As the SNR decreases (i.e., as the α decreases or R increases), the mean and standard deviation increase. The standard deviation showed the same tendency as predicted. The standard deviations from bipolar readouts are lower than those from monopolar readouts, particularly with a shorter TE range, lower α, and higher R.

**Figure 4:**
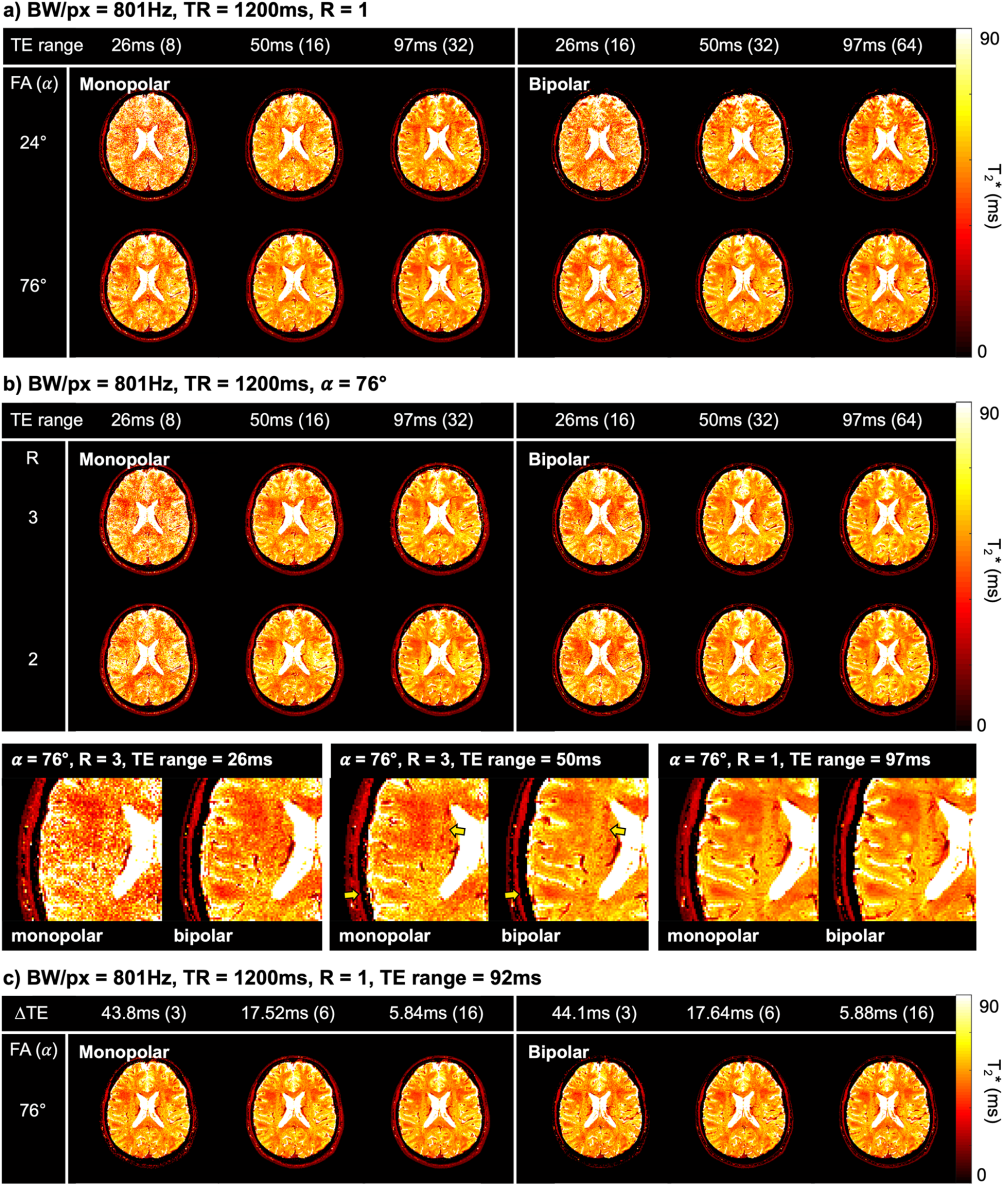
T2* maps from a healthy volunteer obtained using monopolar and bipolar readout gradients with amplitude modulation correction. Each column and row display the results for a different set of (**a**) α and TE range and (**b**) R and TE range. Imaging parameters: BW/px = 801, ∆TE = 2.92 ms for monopolar readout and 1.47 ms for bipolar readout. The number in parentheses is the maximum number of echoes achieved in the given TE range. As indicated by the yellow arrows in the magnified view, anatomical structures are clearly distinguished in the T2* maps from bipolar readouts. The figures in (**c**) show the results for a different set of ∆TEs.

Figure 4 shows the results of in vivo experiments. Figure 4a,b show that the variation in the T2* map increases with an increase in R and a decrease in the α and TE range. The T2* maps from bipolar readouts show less variation than those from monopolar readouts under the same conditions, especially for a higher R and shorter TE range. In addition, T2* maps from bipolar readouts clearly show the anatomical structures. The T2* maps at the bottom of Fig. 4b are magnified maps obtained with an α of 76°. The sulci, gyri, and corona radiate are better distinguished in the T2* map from the bipolar readout than in the T2* map from the monopolar readout, especially for a higher R and shorter TE range, as indicated by yellow arrows. Figure 4c shows that the variation in the T2* map decreases as the ∆TE decreases. The mean and standard deviations were measured in white matter and are shown in Table 3a–c. Table 3d shows the values averaged across all subjects when α was 76°, and the TE range was 97 ms with an R of 1. The measured values are well matched with other literature^3,32,33^.

**Table 3:** Mean, standard deviation, and relative difference of the standard deviation from one healthy volunteer with respect to the (a) α and TE range, (b) R and TE range and (c) ∆TE.

TE range		26 ms	52 ms	97 ms
(a) BW/px = 801 Hz, TR = 1200 ms, R = 1				
24°	Mono	55.95 ± 23.15 (15.96)	53.26 ± 10.23 (6.05)	51.36 ± 8.38 (3.47)
	Bipolar	53.4 ± 16.25 (11.66)	52.5 ± 9.7 (5.2)	51.07 ± 8.67 (2.53)
76°	Mono	51.34 ± 9.5 (6.35)	52.22 ± 6.97 (2.82)	51.64 ± 6.67 (1.6)
	Bipolar	50.99 ± 8.51 (4.49)	52.21 ± 6.8 (2.11)	51.6 ± 6.48 (1.14)
(b) BW/px = 801 Hz, TR = 1200 ms, α = 76°				
R = 3	Mono	54.13 ± 17.36 (11.89)	53.14 ± 9.37 (5.26)	53.36 ± 7.59 (2.97)
	Bipolar	51.46 ± 9.66 (8.3)	52.64 ± 7.32 (3.88)	52.4 ± 6.9 (2.1)
R = 2	Mono	53.05 ± 12.72 (8.51)	53.22 ± 8.35 (3.8)	52.81 ± 7.38 (2.1)
	Bipolar	51.63 ± 9.76 (6.02)	52.77 ± 7.42 (2.8)	52.51 ± 6.92 (1.51)

∆TE (mono/bipolar)		43.8/44.1 ms	17.52/17.64 ms	5.84/5.88 ms
(c) BW/px = 801Hz, TR = 1200ms, TE range = 92ms, R = 1				
75°	Mono	51.46 ± 7.64 (4.32)	51.55 ± 7.14 (4.19)	51.69 ± 6.84 (2.81)
	Bipolar	51.37 ± 7.59 (4.4)	51.38 ± 7.03 (3.34)	52.57 ± 6.69 (2.24)

	Monopolar	Bipolar	Literatures	
(d) BW/px = 801Hz, TR = 1200ms, α = 76°, R = 1, TE range = 97ms				
ROI	Mean ± std	Mean ± std	–	
Grey matter	58.94 ± 0.96	58.85 ± 0.89	55.7^32^, 65.7^3^	
White matter	50.59 ± 1.05	50.53 ± 1.07	50^32^, 47.6^3^	
Caudate	50.43 ± 0.95	50.57 ± 0.7	54.9^3^	
Pallidus	33.98 ± 3.89	33.35 ± 3.92	30^33^	
Putamen	45.35 ± 1.23	45.17 ± 0.94	41^33^	
Thalamus	50.29 ± 0.74	50.27 ± 0.47	50^33^	

The values were measured in white matter. The numbers in parentheses are the standard deviations predicted from CRLB. As in the computer simulation and phantom experiments, standard deviation increases as the α and TE range decrease. (a and b) The difference between both readouts also tends to increase with a decrease in the α and TE range. (c) When monopolar and bipolar readouts have similar TEs, both readouts show comparable precision. (d) The mean and standard deviation averaged across all subjects (number of subjects = 2). The values were calculated from the data with α of 76° and R of 1 for the maximum TE range (i.e., 97 ms).

**Figure 5:**
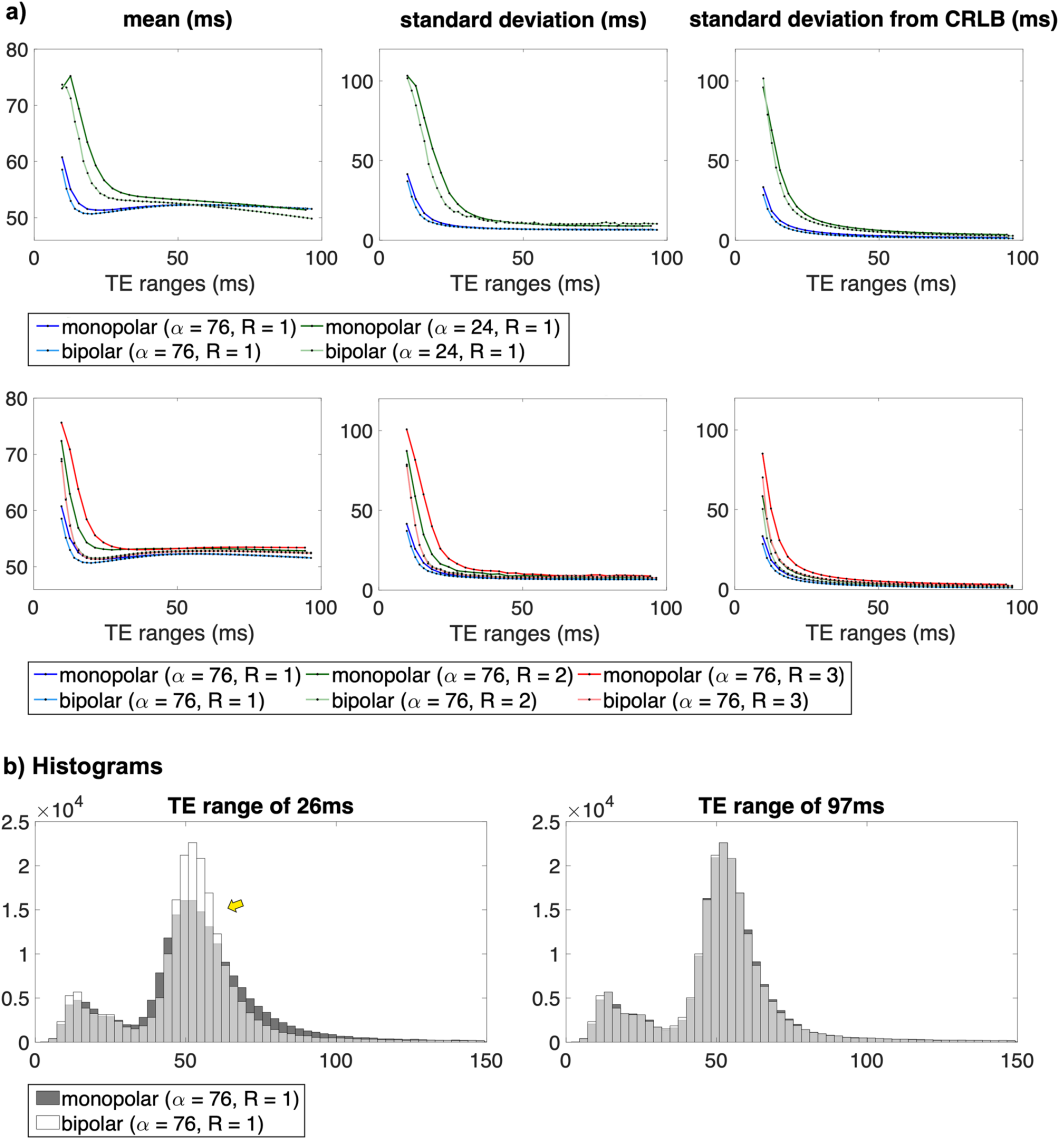
(**a**) Plots of the mean and standard deviation of the T2*s calculated in white matter from one healthy volunteer. Imaging parameters: BW/px = 801, ∆TE = 2.92 ms for monopolar and 1.47 ms for bipolar, and TR = 1200 ms. (**b**) Histograms for the whole brain when the TE range is 26 ms (left) and 97 ms (right), respectively. Imaging parameters: BW/px = 801, ∆TE = 2.92 ms for monopolar and 1.47 ms for bipolar, TR = 1200 ms, α = 76°, R = 1.

Figure 5a shows the plots of the mean, standard deviation and CRLB values calculated in white matter as a function of the TE range. The measured values show the same tendencies as in the results of the computer simulation and phantom experiments. The mean and standard deviation decrease as the α and TE range increase and the R decreases. The mean and standard deviation from both readouts are almost identical for the maximum TE range. As the TE range decreases, the bipolar readout tends to achieve a lower standard deviation compared to the monopolar readout. Figure 5b shows histograms for the whole brain when the TE range is 26 ms and 97 ms with an α of 76° and R of 1. The histograms of monopolar and bipolar readouts are represented in black and white. The grey region indicates the intersection of both readouts. When the TE range is 26 ms, it can be observed that the width of the bipolar readout is smaller and the peak is higher than that of the monopolar readout, i.e., bipolar readout provides greater precision.

## Discussion

In this work, the effects of imaging parameters, i.e., α, TR, TE range, ∆TE and BW, on T2* quantification were investigated, and monopolar and bipolar readouts were compared using computer simulation, phantom and in vivo experiments. Initially, datasets were acquired for a different set of α and TRs to investigate the impacts of noise. Since the multi-echo GRE sequences employ a multi-slice acquisition to utilise the dead time, the TE range is determined by the TR and the number of slices, *N*_*s*_. The reduction in the TR with a given *N*_*s*_ not only limits the *N*_*e*_ but also causes a decrease in the SNR. Thus, in order to avoid mixing the two effects, the *N*_*e*_ remained fixed in the first experiments. For a given α and TR, the *N*_*e*_ can be increased by reducing the *N*_*s*_. The second set of experiments (i.e., select *n* echoes out of the echoes acquired) indirectly shows the situation when the TR is kept constant, but *N*_*s*_ is altered in a multi-slice acquisition. In the third set of experiments, every *n*-*th* echo out of the echoes acquired was retrospectively selected to see the effects of ∆TE. In general, an effort is made to minimise ∆TE (i.e., no time is wasted between readout gradients and/or increase the BW) and to acquire more echoes to aid accurate T2* quantification. However, there is a trade-off between ∆TE and BW. An increase in the BW reduces ∆TE but decreases the SNR. These effects on T2* quantification were observed in the fourth set of experiments by changing the BW.

As T2* was a known value in the computer simulations, it was possible to use the computer simulations to investigate the effects the imaging parameters had in terms of accuracy and precision, thus facilitating the evaluation of the quantification. In the simulation, the time-series signals were modelled as a mono-exponential function since the phantom used in our experiments was quite large and homogenous. From the simulation results, it was found that the mean T2* deviated from the true value for a shorter TE range and reached the true value as the TE range increased (Fig. 3). If the TE range is sufficient, even when the number of echoes is limited by reducing the ∆TE (Table 1c,d), a mean T2* close to the true value could be delivered. This implies that signal acquisition over a sufficient TE range contributes more to an accurate quantification compared to increasing the number of echoes while having a shorter TE range. Figure S2 in Supplementary Information shows the changes in mean T2* with respect to the TE range for different ROIs. To achieve a T2* similar to the true value, the TE range should be comparable to or greater than the true T2*. The difference in the mean values between the two readout types was less than 0.1% for a sufficient TE range, i.e., monopolar and bipolar readouts have almost the same accuracy. When the TE range was shorter than true T2*, the mean from the bipolar readout was found to be closer to the true value than that from the monopolar readout, i.e., the bipolar readout has higher accuracy for a relatively shorter TE range. The mean T2* increased as the SNR or TE range decreased. When additional noise or a short TE range is introduced, the fitting procedure is more likely to be trapped at the erroneous local minima. The tendency of T2* to increase might be interpreted as a result of an increase in the number of outliers. The outliers are related to poor initial estimates. In our work, the Rician noise distribution was considered in a fit model^28,29^ because noise bias can induce an overestimation of T2*^35–37^. As the noise was estimated using a ROI in the background region, this approach could also yield erroneous noise estimates due to spatially varying noise fields, which may be a limitation of this study.

In terms of precision, a remarkable difference between the two readouts can be seen; the bipolar readout always provided greater precision (i.e., lower standard deviation) compared to the monopolar readout. The difference between the standard deviations of the two readouts was over 25%, regardless of the TE range, with a BW/px of 801 (Table 1b). The bipolar readout utilises twice as many echoes as a monopolar readout in a given TE range. In theory, the standard deviation of T2*s from a bipolar readout is √2/2 times that from a monopolar readout. The standard deviations predicted from CRLB showed a √twofold difference between the monopolar and bipolar readouts. The standard deviations measured had similar values to the CRLB values in both readouts and showed a √twofold difference, particularly for a higher α and longer TE range. As α and TE range decreased, the difference between the two readouts became greater than √2. The measured standard deviations showed good agreement with the CRLB values, except for the short TE range (< = 26 ms) and lower α. As with the changes in the mean T2*, outliers caused by insufficient SNR or data points could result in an increase in the standard deviation.

In the phantom experiments, the differences in the standard deviation between monopolar and bipolar readouts were considerably reduced for a higher α and longer TE range. This was also confirmed in the in vivo experiments. In the presence of systematic or physiological errors, both readouts provided similar accuracy and precision, especially when the signal was sufficiently sampled (i.e., with a relatively longer TE range). However, the bipolar readout showed smaller standard deviations than the monopolar readout as the TE range decreased. The range of variation (i.e., the difference between the highest and lowest means or standard deviations) increases as the relaxation time increases (Fig. S3 in the Supplementary Information). The bipolar readouts were less sensitive to changes in the R, TE range and relaxation time.

An increase in the BW can shorten the ∆TE. The difference in the *N*_*e*_ between the two readouts increases as the BW increases. In practical implementations, the ∆TE is constrained by the maximum amplitude and slew rate of the gradient. If a high-performance gradient is utilised, a bipolar readout can be more robust for T2* quantification. In addition, the range of T2* relaxation times investigated in this study is specific to the brain at 3 T. If T2* quantification is performed with a short TE range in an area with a short T2* value, such as the liver, it is expected that the benefit of using a bipolar readout would become more apparent.

Amplitude modulation, like phase modulation, produces a linear difference in the signal intensities and is dependent on the polarity of the readout gradients. When a monopolar readout is used, the modulation term appears identically throughout the echo train, having no significant influence on T2* quantification. However, T2* quantification would be hampered by amplitude modulation in the bipolar readout. In our experiments, when bipolar readouts were used, fluctuations in the mean T2* were observed across the TE ranges. The effects of amplitude modulation were also visible in uncorrected T2* maps. The quantified T2* showed differences between the left and right sides of the object. The observed decrease in fluctuations in the mean T2* as the TE range increases also suggests that the impact of amplitude modulation increases when the signal is not sufficiently sampled due to a relatively shorter TE range. However, the fluctuations can be suppressed by an amplitude modulation correction. Thus, it is important to note that amplitude modulation correction is required to obtain accurate T2*, especially for a shorter TE range.

## Conclusions

In this work, the performance of monopolar and bipolar readout gradients was evaluated under various conditions. A shorter ∆TE in the bipolar scheme enabled the utilisation of a larger number of echoes, which led to the more accurate quantification of T2*. Moreover, the bipolar readouts were shown to be more robust in quantifying the T2* when a lower SNR or smaller number of echoes was given. In the case of using bipolar readout, the effect of amplitude modulation was demonstrated, particularly for a shorter TE range. Consequently, the use of bipolar readout gradients would be advantageous for accurate T2* quantification with amplitude modulation correction.

## Supplementary Information

Supplementary Information.

## Data Availability

The datasets used and /or analysed in the current study can be shared by submitting a formal request to the corresponding author, N. Jon Shah (n.j.shah@fz-juelich.de). The in vivo data and metadata are protected under the ethics/internal administrating documents.

## Abbreviations

GRE: Multi-echo gradient echo
EPI: Echo-planar imaging
CRLB: Cramér-Rao lower bound
SNR: Signal-to-noise ratio
TE: Echo time
TR: Repetition time
ROI: Region of interest
BW: Bandwidth
