# Supplementary material for: T2* quantification using multi-echo gradient echo sequences: a comparative study of different readout gradients
Source: Sci Rep. 2023 Jan 20;13:1138. doi: 10.1038/s41598-023-28265-0 (PMC9860026; doi:10.1038/s41598-023-28265-0)
Supplement: Supplementary file 1 — Supplementary Information. [file 41598_2023_28265_MOESM1_ESM.pdf]

Supplementary Information Figure Legends & Figures

Figure S1.

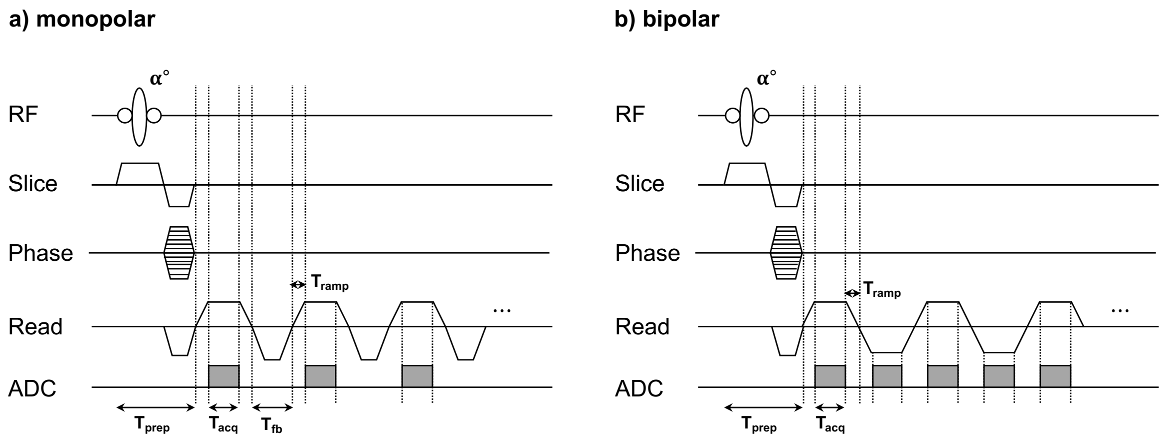

Figure S1. Schematic diagrams of multi-echo GRE using (a) monopolar and (b) bipolar readout gradients. In monopolar readout, fly-back gradients are inserted between echoes. All echoes are obtained with positive readout gradients. In contrast, for bipolar readout, multiple echoes are obtained using both positive and negative readout gradients.

Table S1.

a) first simulation: effects of α and TR

the number of samples for each echo (N_x)	240
receiver bandwidth (BW)	192.24kHz (BW/px = 801Hz)
TE range	43ms
TE_1	3.84ms
∆TE	2.92ms for monopolar (N_e = 14)<br>1.47ms for bipolar (N_e = 27)
TR	600, 900, 1200ms
flip angle (α)	15°, 35°, 75°
A total of datasets for each readout	9 (three αs × three TRs)

b) second simulation: effects of TE range

the number of samples for each echo (N_x)	240
receiver bandwidth (BW)	192.24kHz (BW/px = 801Hz)
TE range	97ms
TE_1	3.84ms
∆TE	2.92ms for monopolar (N_e = 32)<br>1.47ms for bipolar (N_e = 64)
TR	1200ms
flip angle (α)	15°, 35°, 75°
A total of datasets for each readout	3 (three αs)<br>→ retrospective subsampling<br>1) first *n* echoes 2) every *n-th* echoes out of the echoes acquired were subsampled

c) third simulation: effects of BW

the number of samples for each echo (N_x)	240
receiver bandwidth (BW)	82.08kHz (BW/px = 342Hz)
TE range	97ms
TE_1	4.62
∆TE	4.48ms for monopolar (N_e = 32)<br>3.03ms for bipolar (N_e = 64)
TR	1200ms
flip angle (α)	75°

the number of samples for each echo (N_x)	240
receiver bandwidth (BW)	147.12kHz (BW/px = 613Hz)
TE range	97ms
TE_1	4.01ms
∆TE	3.27ms for monopolar (N_e = 32)<br>1.8ms for bipolar (N_e = 64)

TR	1200ms
flip angle (α)	75°

the number of samples for each echo (N_x)	240
receiver bandwidth (BW)	172.32kHz (BW/px = 718Hz)
TE range	97ms
TE_1	3.9ms
∆TE	3.05ms for monopolar (N_e = 32)<br>1.6ms for bipolar (N_e = 64)
TR	1200ms
flip angle (α)	75°

Table S1. Imaging parameters used in the (a) first, (b) second, and (c) third computer simulations. The values of T_{prep} = 3.1ms, T_{fb} = 1.45ms, M_0 = 1, φ_0 = 0°, Δf = 10Hz, N_x = 240, σ_0 = 0.0128, BW_0 = 192.24kHz were used. The T_{ramp} was calculated by subtracting T_{acq} (or T_{acq}+ T_{fb} for monopolar) from the ΔTE actually available at the scanner. The N_e represents the total number of echoes.

Figure S2.

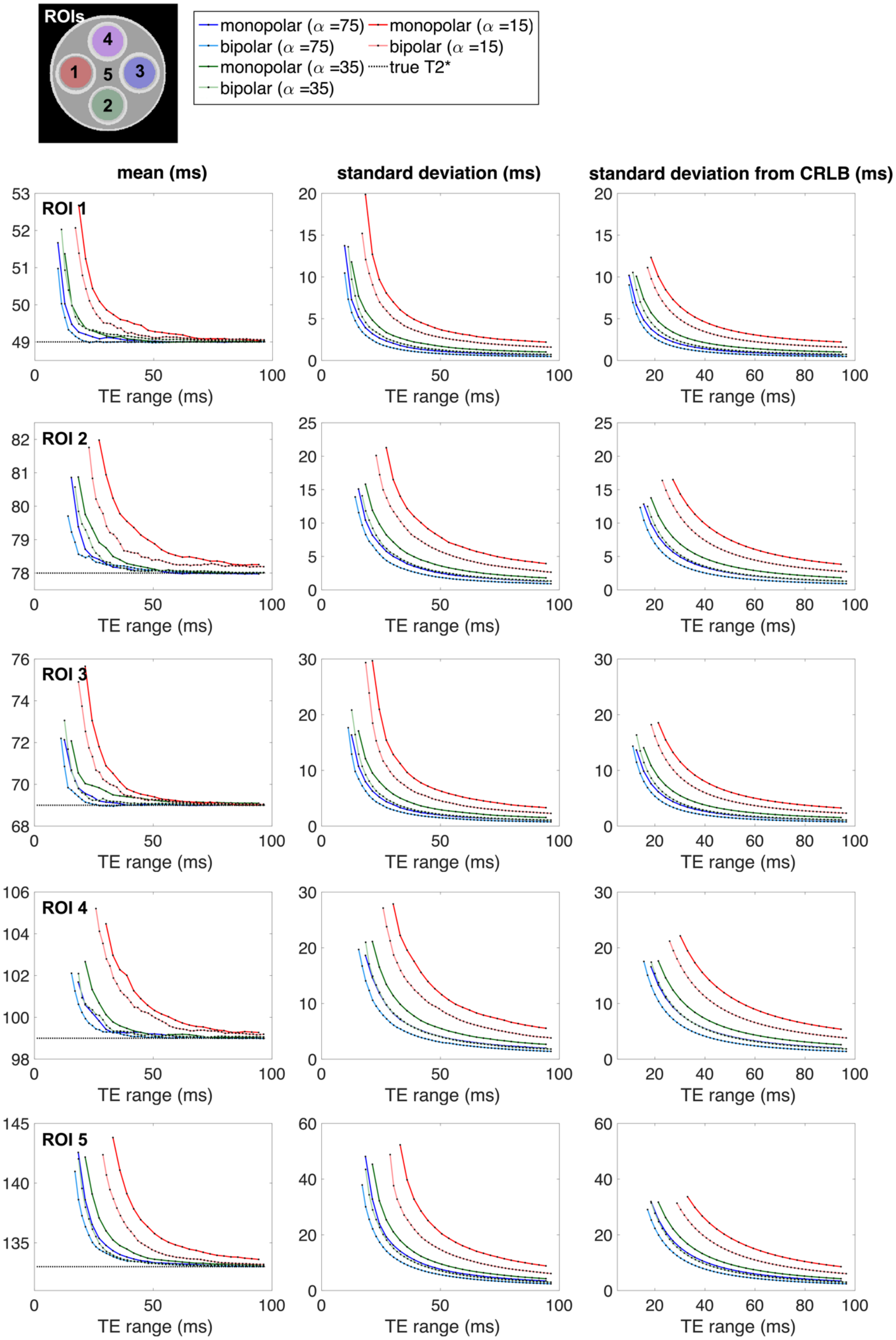
ROIs
1
2
3
4
5
monopolar (α =75)
bipolar (α =75)
monopolar (α =35)
bipolar (α =35)
monopolar (α =15)
bipolar (α =15)
true T2*
mean (ms)
standard deviation (ms)
standard deviation from CRLB (ms)
ROI 1
ROI 2
ROI 3
ROI 4
ROI 5
TE range (ms)

Figure S2. Plots of the mean and standard deviation of the calculated T2*s and the standard deviation predicted from CRLB in computer simulation as a function of TE range. Each row shows the results calculated from each ROI. Imaging parameters: BW/px = 801, ΔTE = 2.92ms for monopolar and 1.47ms for bipolar, TR = 1200ms. The αs of 75°, 35°, and 15° are denoted by blue, green, and red lines, respectively. The results from monopolar and bipolar settings are displayed with dark and light colours, respectively.

Figure S3a

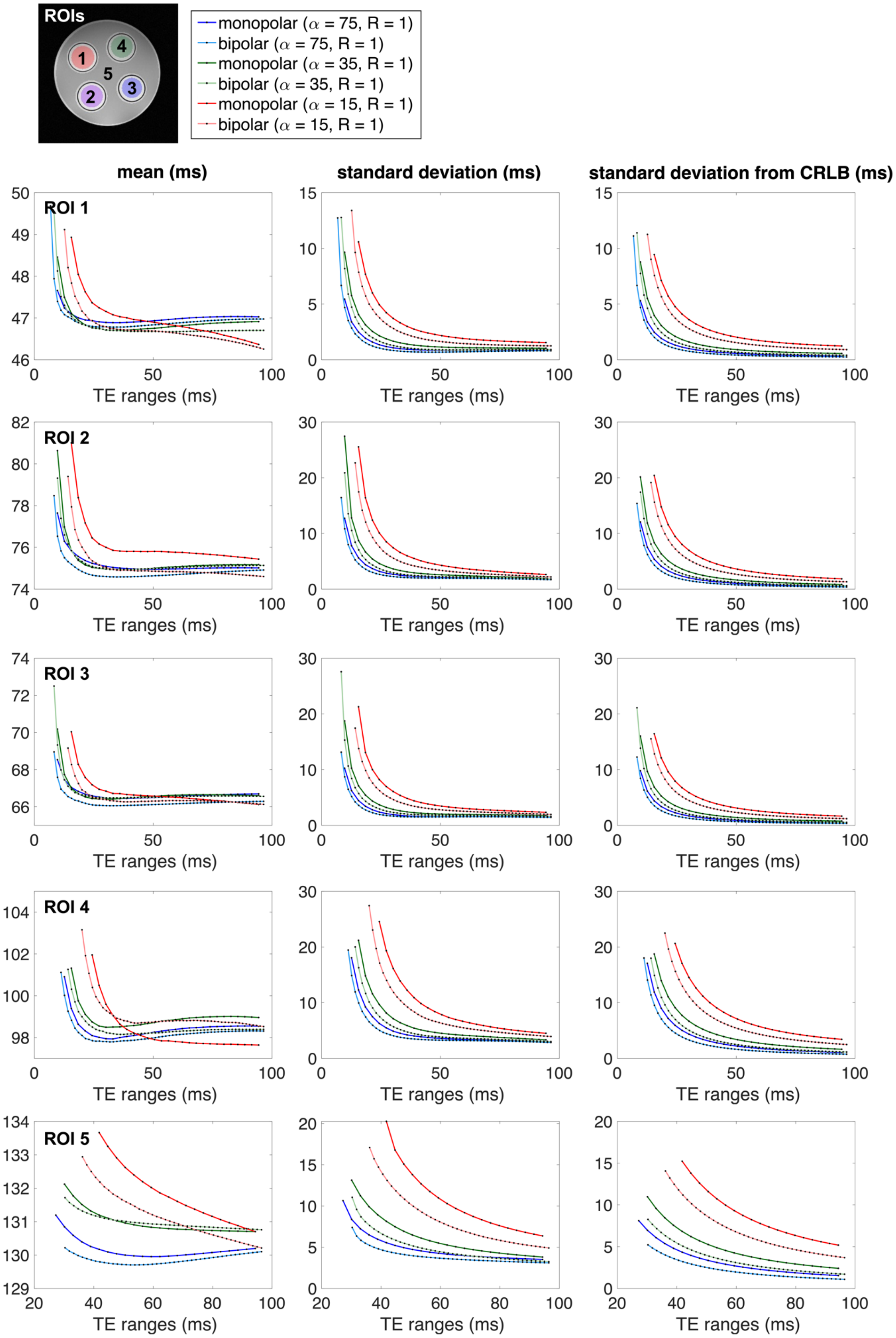

ROIs
1
2
3
4
5
monopolar (α = 75, R = 1)
bipolar (α = 75, R = 1)
monopolar (α = 35, R = 1)
bipolar (α = 35, R = 1)
monopolar (α = 15, R = 1)
bipolar (α = 15, R = 1)
mean (ms)
standard deviation (ms)
standard deviation from CRLB (ms)
ROI 1
ROI 2
ROI 3
ROI 4
ROI 5
TE ranges (ms)

Figure S3b

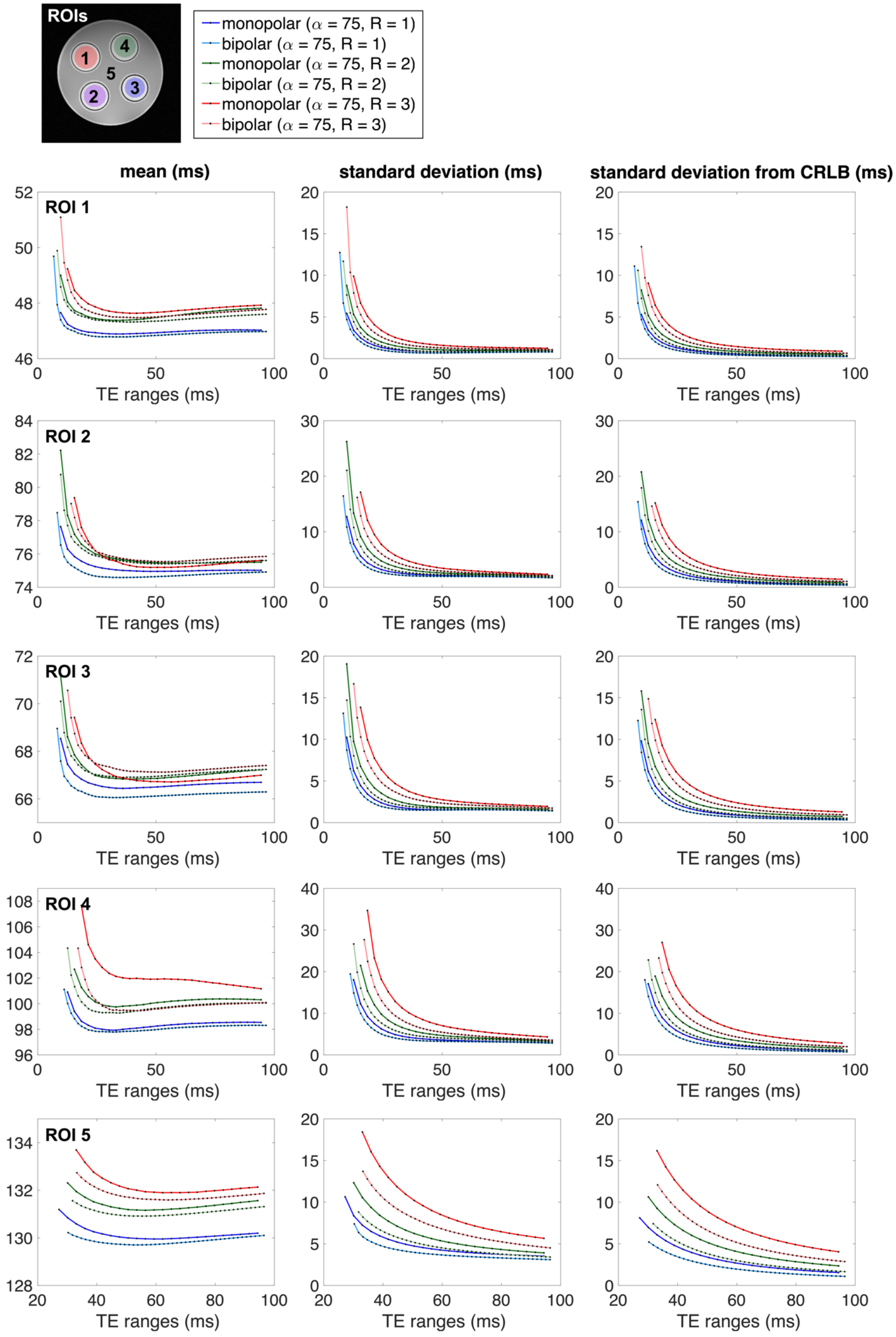

ROIs
1
2
3
4
5
monopolar (α = 75, R = 1)
bipolar (α = 75, R = 1)
monopolar (α = 75, R = 2)
bipolar (α = 75, R = 2)
monopolar (α = 75, R = 3)
bipolar (α = 75, R = 3)
mean (ms)
standard deviation (ms)
standard deviation from CRLB (ms)
ROI 1
ROI 2
ROI 3
ROI 4
ROI 5
TE ranges (ms)

Figure S3. Plots of the mean and standard deviation of the calculated T2*s from phantom experiments and the standard deviation from CRLB as a function of the TE range. The changes in mean and standard deviation are displayed with respect to the (a) α and (b) R. Imaging parameters: BW/px = 801, ΔTE = 2.92ms for monopolar and 1.47ms for bipolar, TR = 1200ms. In (b), the α is 76°.
